# Embedded health service development and research: why and how to do it (a ten-stage guide)

**DOI:** 10.1186/s12961-018-0344-7

**Published:** 2018-07-25

**Authors:** John Walley, Mohammad Amir Khan, Sophie Witter, Rumana Haque, James Newell, Xiaolin Wei

**Affiliations:** 1 Leeds Institute of Health Sciences, University of Leeds, England, United Kingdom; 2 Association for Social Development, Islamabad, Pakistan; 3 Institute for Global Health and Development, Queen Margaret University Edinburgh, Musselburgh, Edinburgh EH21 6UU United Kingdom; 4 University of Dhaka, Dhaka, Bangladesh; 5 Dalla Lana School of Public Health, University of Toronto, Toronto, Ontario Canada

**Keywords:** Health services, Embedded development and research, Operational research, Research into practice, Knowledge co-production, Intervention scale-up

## Abstract

In a world of changing disease burdens, poor quality care and constrained health budgets, finding effective approaches to developing and implementing evidence-based health services is crucial. Much has been published on developing service tools and protocols, operational research and getting policy into practice but these are often undertaken in isolation from one another. This paper, based on 25 years of experience in a range of low and middle income contexts as well as wider literature, presents a systematic approach to connecting these activities in an embedded development and research approach. This approach can circumvent common problems such as lack of local ownership of new programmes, unrealistic resource requirements and poor implementation.

We lay out a ten-step process, which is based on long-term partnerships and working within local systems and constraints and may be tailored to the context and needs. Service development and operational research is best prioritised, designed, conducted and replicated when it is embedded within ministry of health and national programmes. Care packages should from the outset be designed for scale-up, which is why the piloting stage is so crucial. In this way, the resulting package of care will be feasible within the context and will address local priorities. Researchers must be entrepreneurial and responsive to windows of funding for scale-up, working in real-world contexts where funding and decisions do not wait for evidence, so evidence generation has to be pragmatic to meet and ensure best use of the policy and financing cycles. The research should generate tested and easily usable tools, training materials and processes for use in scale-up. Development of the package should work within and strengthen the health system and other service delivery strategies to ensure that unintended negative consequences are minimised and that the strengthened systems support quality care and effective scale up of the package.

While embedded development and research is promoted in theory, it is not yet practiced at scale by many initiatives, leading to wasted resources and un-sustained programmes. This guide presents a systematic and practical guide to support more effective engagements in future, both in developing interventions and supporting evidence-based scale-up.

## Background

In a world of growing and changing disease burdens and constrained health budgets, finding effective approaches to developing and implementing evidence-based health services is crucial, and underpins the Sustainable Health Goal target of Universal Health Coverage [1]. Much has been published on evidence review and developing guidelines [2],operational and implementation research [3], knowledge brokering [4–6], getting policy into practice [7–9], and on embedded research [10] but these are often described and undertaken in isolation from one another. Moreover, much of the literature is based on higher income country experiences. This paper, which is based on 25 years of experience in developing and strengthening service delivery for communicable and non-communicable diseases in low and middle income countries, presents a systematic approach to connecting these activities in an embedded development and research approach.

Embedded development and research is here used to indicate two important but distinct aspects: first, that all stages of the process are co-produced by researchers (local and international) and local programme managers, working in close partnership but bringing different skills, insights and connections; and secondly, that the intervention and associated research are closely tailored to local needs and resources (in other words, are themselves well embedded as part of the development process). This can circumvent common problems such as lack of local ownership of new programmes, unrealistic resource requirements and poor implementation [11]. This approach can be broken down into ten steps, grouped into four stages, which are described below (with an example from one area of work given in Box 1). While the steps may be familiar, they are rarely undertaken systematically and sequentially. We believe that following this step-wise approach greatly improves the chance of health services being of high quality, universal, effective and sustained, even in challenging low and middle income contexts.

## Ten steps

### Designing and developing the service delivery package

Research into a weak, poorly contextualised or unsustainable intervention is a waste of precious time and resources, which is why our process begins with a careful design and development stage. This typically comprises four steps: (1) assess the problem; (2) identify options for addressing them; (3) carry out additional exploratory research as required; and (4) develop locally adapted tools and service guidelines.

#### 1. Assess the problem

The intervention and its associated guidelines and tools should be designed according to the best evidence available and in line with the local context. The process therefore starts with a review, in collaboration with local programme managers and policy-makers in Ministries of Health, of existing literature on the problem’s distribution and underlying causes, as well as the experiences of earlier programmes there and elsewhere (barriers to service delivery and user access, what has been learned about addressing them, resource requirements etc.) [12]. Where researchers, local and international, work in a long-term partnership with programmes and ministries, they already know the context and assessment can be tightly focussed on the new potential intervention – involving, for example, rapid data collection with a checklist at a few facilities to identify bottlenecks and opportunities.

#### 2. Identify options for addressing the problem

This step involves a review of the best technical interventions for the disease or condition, often based on systematic reviews (for example, [1, 13, 14], original research papers, programme reports and guidelines from the World Health Organisation or other international development organisations, as relevant. Evidence from these can be presented in an adaptation guide (for example, [15]), which includes the options and evidence relevant for the specific country setting.

#### 3. Carry out additional exploratory research

Where relevant information is lacking, such as on local health behaviours, exploratory research should be carried out to inform and refine the intervention. For example, there may be a need to better understand the beliefs and practices of clients and service providers relating to the disease or condition (for example, [2, 3, 16]. Gathering this information early in the process will help to ensure that the guides, tools and case studies used in training are adapted to take into account prevalent beliefs, practices and systems [17, 18].

#### 4. Develop a package of guidelines and tools adapted to the local context

Based on the previous steps, the research team can now develop a package that incorporates current knowledge on effective interventions and that includes practical guidelines and tools [19]. It should designed to be effective and feasible within the country health service context – i.e. evidence-based and high-quality, but also taking into account the way that health facility and district health systems are configured, as well as the resources which are available (such as staff time, funding and equipment). The package may be used as a vehicle to introduce research-informed approaches – for example new diagnostic procedures, drug regimens, changes to organisation or processes of care, treatment support and training methods – into practice at scale.

The intervention should aim to integrate the intervention within, and strengthen, the existing health system. This includes necessary supportive health system strategies, such as managing medications and other supplies, assuring quality of laboratory testing, staff training, referral pathways, and performance monitoring and supervision, all of which should be included in the guides and tools.

The national programme may already have a guideline providing broad policy, drug regimens, roles and responsibilities. These are necessary, but not generally sufficient. The package of care to be developed often needs to include the following:

- User-friendly operational guides to meet the needs of managers (for example, district implementation planning, supervision, performance management and community intervention guides)
- Case management desk guides (concise, practical and role-specific handbooks) for doctors, nurses and paramedics to use during patient consultations. These follow each step of the care process, including identification, diagnosis, education and follow-up. The guide and tools may be used by public, non-governmental organisation and private providers or educators.
- For community health workers, simple and concise case management and health education tools, often called “job aids”
- Tools including treatment cards, registers, patient education flipcharts and leaflets and training modules for health workers.

Adaptation and development of the package can be organised around three steps [20]:

1. Initially the package is “pre-edited” by one or more health professionals experienced in the country, specific disease and service delivery context.
2. Next a technical working group is formed. This should consist of around 6 people, familiar with the content and setting, who can work around a table. Representation should include relevant programme managers, care providers, technical partners (, such as the World Health Organisation, UNICEF, other non-governmental partners), and clinical and intervention development experts. They should understand the public health approach requiring standardisation and be able to simplify procedures so that these will be feasible when scaled up. They should not assume, for example, that many lab tests and treatments used in tertiary hospitals will be available in primary care. They should also ensure that local terminology, suitable for health managers and facility staff, is used. They should meet weekly or attend a 2–3 day workshop and should review and edit the pre-edited materials section by section. Comments and revisions can also be collected prior to reduce the time of the workshop. During the workshop, particular issues should be discussed, and changes agreed, while the facilitator notes down what requires further editing. Good communication within the group is essential.
3. Lastly a steering committee including senior decision-makers reviews and endorses the use of the materials.

Typical types of adaptation may include:

- Adapting operational strategies to deliver each care or intervention task;
- Editing treatment regimens according to national guidelines;
- Adding or amending treatment record cards and registers;
- Developing health education, lifestyle and adherence messages for the specific disease, and adherence support strategies e.g. by family members, volunteers or by mobile phone, with follow-up care in the health centre.

It is vital that interventions are designed from the start to be feasible, sustainable and widely replicable.

### Pre-test and pilot the package

This stage is critical in road-testing the package in real contexts, ironing out problems and carrying out a second wave of adaptations as needed to make it feasible and acceptable. If a rapid decision is needed on whether to scale up, this stage, with its accompanying small-scale research, can be sufficient to inform robust implementation and scale-up.

#### 5. Pre-test the guides and tools

Pre-testing commonly involves reviewing the contents of drafted materials, with a group of selected care providers and programme staff, getting their feedback on the appropriateness of the content (i.e. adequacy, correctness, acceptability etc.) and revising as necessary. The purpose of pre-testing is to ensure tools do what they are supposed to do e.g. a picture conveys the intended message; a role play exercise teaches the individual the intended skill; a register records the intended information.. Pre-testing should be done before piloting with real patients or in a community intervention [20, 21].

#### 6. Pilot

Piloting, also called feasibility assessment, involves trying the package out at small scale to assess feasibility and acceptability, and if necessary making revisions. It is important to try out the new intervention under routine conditions to test the feasibility of the delivery strategy.

Piloting should be in an accessible district, selecting a few health facilities or villages. A training course is run with relevant health workers. The facilitators take turns running the sessions and annotating the guides and module content to improve clarity. The intervention, including the supervision and monitoring, is implemented for some months in the chosen pilot sites. During the pilot, health workers record (using the proposed treatment cards and registers and community outreach records as relevant) data on the care process. The staff adherence to the care protocols is assessed through review of patient records, supported (if possible) by observation and interviewing, whereas the client adherence to follow-up visits is assessed from ‘drop-outs’ through each stage of the process [22].

The package may also be tested using one or more qualitative methods, such as interviews, group discussions, observation of use and exit interviews. Studies assess the feasibility and acceptability of the intervention package from the perspective of the service providers and users. The intervention and study can then be improved based on the findings of the pilot [20].

Unfortunately, “piloting” has obtained a bad reputation because commonly projects do not get beyond the pilot stage, often because the intervention has not been designed to be sustainable and replicable. For example, a well-funded pilot may achieve good results which are not sustained under normal programme funding; or a pilot supported by project team time achieves good results, but scale-up fails when similar support is not available nationwide. (Such pilots should more accurately be termed demonstration sites.) The carefully designed and implemented pilot is an opportunity to refine the package *under routine conditions*, with the procedures, guides and tools corresponding with the human, material and financial resources that will realistically be available during scale-up.

### Implement and evaluate the intervention

Implementation and financing of the package should be led by the national programme or Ministry to ensure that it is technically and financially sustainable. If there is interest, time and funds, larger scale trials, economic evaluations and process evaluation can accompany the implementation, as described below.

#### 7. Evaluate effectiveness using an embedded trial

In embedded research we often evaluate as part of phased programme implementation. It is best for programmes not to attempt to implement everywhere at once. This may seem obvious, but often programmes instruct all sites nationwide to implement together before they are ready. This results in poor implementation and poor outcomes. Effective implementation requires much care to ensure local procedures are ready. The intervention can then be implemented site by site, systematically scaling up until all sites are covered. Phased implementation provides an opportunity to develop a randomised controlled trial. Trial sites are randomly allocated to early-implementation ‘intervention’ sites, and outcomes compared with those from pre-implementation ‘control’ sites, to estimate the effectiveness of the package relative to the existing service [4, 23–25]. External research support is generally needed to plan and execute a randomised controlled trial [26].

In randomised controlled trials and other controlled evaluations it is important to implement standardised case identification and recording on treatment cards and registers in both the intervention and control sites, also ensuring that all equipment, lab tests and drugs needed are available for all patients. The trial can be of the care package as a whole, with the control sites continuing with ‘usual’ (existing) care [21, 27–29], or of a component of the package such as lifestyle behaviour change [30, 31]. In either case, the elements to be trialled are omitted from the control sites’ guides and tools.

#### 8. Evaluate costs and cost-effectiveness

Costing studies are often conducted alongside evaluation of effectiveness. At a minimum, assessment is of the incremental cost *to the health service* to add the service. This form of economic evaluation estimates the likely costs to the provider to replicate the service, should it be found effective. It is also useful to carry out an incremental cost effectiveness analysis, to estimate the incremental cost per successful treatment (for example, the incremental cost per child using a bednet, per child vaccinated or per successfully-treated TB patient) [32, 33]. It is often important to also estimate changes in costs to *patients* that result from new strategies (e.g. switching from hospital-based to community-based care for drug-resistant tuberculosis) to identify when health service costs are transferred to patients. The cost and cost-effectiveness studies are useful to estimate the cost of scaling up and what implications this will have for national or local health budgets, and can be used to leverage additional programme funding.

#### 9. Evaluate the implementation of various parts of the package

Process evaluations systematically explore what happens within the package, rather than merely looking at the package as a whole. Process evaluation studies may be conducted to understand the “why” of results, for example why the package was (or was not) more effective than usual care [34]. Sites, clinicians and clients are observed and interviewed to find out which parts of the guides and tools were used as intended, and which parts were not (‘fidelity’ to the package), the reasons behind this, and how they can be addressed [34]. The study may assess interventions in terms of ‘how’ the patient, family, and community factors, such as access to and quality of the community intervention or health service, and direct and opportunity costs influence patients’ ability to attend, be diagnosed, treated and adhere to follow-up appointments [5, 35]. As with the pilot qualitative study, this may comprise semi-structured interviews with various categories of patients and carers, and focus groups with family, community members, providers and managers, as appropriate.

### Support policy and practice change

In the embedded research and development approach, scalability of the package is addressed from the start, which enables support to scale up and wider dissemination.

#### 10. Support scale up and wider dissemination

Through all stages, the approach is to develop services that align with national policies and are adapted to be feasible and effective in the local context. Implementation and financing is led by the programme rather than researchers. There is collaboration between researchers and the programme managers and service providers at different levels of the health system (as relevant, based on the nature of the intervention) to develop, adapt, pilot and roll out the package. In this final stage, all parties discuss the research findings, informing further changes to guides and services. National scale-up of the package by the national programme should initially be with support from local and international researchers. Researchers need to actively look for any policy opportunities that can embed the interventions to promote wider dissemination at the system level.

In relation to planning and financing, joint actions at this stage include:

- Obtaining decision maker approval, which may require drafting briefing documents and preparing presentations
- Integrating the intervention in programme plans, which may require drafting or editing documents, facilitating discussions, and making presentations
- Mobilising resources from public and donor sources, which may require drafting of proposal(s), responding to queries, and revising proposals in response to comments

Implementation activities commonly include:

- Printing the finalised materials for use in pre- and in-service courses and on-going service delivery
- Rolling out training, including teaching the skills to use these materials through role-play exercises
- Ensuring drugs, cards, registers and laboratory materials are available in time so that health workers can immediately put into practice what they learnt in training
- Follow-up after training, including supervisory visits to each participant to observe and give encouragement, both as they start to use the guide and tools, and systematic routine monitoring of performance [36].

The learning and the products are disseminated both in the country and internationally (though the usual channels such as meetings, presentations, project briefs and peer-reviewed publications). The intervention and package of care can be edited to become a non-country-specific ‘generic’ version, available for adaptation in other countries, following the stages of service development and research outlined above [20].

## Conclusions

We describe in this article a set of ten steps which have been found to work well in a range of low and middle-income countries in Africa and Asia over a 20-year period in delivering scaled-up, evidence-based health service programmes at different levels of the health system which bring important health benefits for communities. This is not a rigid process and should be tailored to the context and needs. However, it is important to understand and respect the underlying approach, which is based on long-term partnerships and working within local systems and constraints. Service development and operational research are best prioritised, designed, conducted and replicated when embedded within Ministry of Health and national programmes. In this way, packages of care will address local needs and be feasible there. There is no benefit to testing an infeasible package, yet commonly, researchers test ‘ideal’ but impractical strategies, then seek to ‘market’ their results to policy-makers. Care packages should from the outset be designed for scale-up, which is why the development and piloting stages are so crucial.

Development should be accompanied by research, which informs it in an iterative way, with scale of research tailored to the level of importance of the questions and the available resources and time. Researchers must be responsive to windows of funding for scale-up (domestic and international, such as global funds for AIDS, TB, malaria), working in real-world contexts where funding and decisions do not wait for evidence, so evidence generation has to be pragmatic and timely to meet and ensure best use of the policy and financing cycles. This requires policy entrepreneurial skillsets which may not have been in the traditional researcher toolbox, as well, of course, as the ability to foster and deliver robust research and work with partners from the health provider level up to Ministry of Health, and including public and private sectors. Some of these organisations present difficult environments for evidence-based programming, with limited time and incentives [37]; however, skilled identification of champions and windows of opportunity, as well as sheer perseverance, can allow for success. The development and research go together, in order to generate tested, practical and easily usable tools and training materials and processes for use in scale-up. In low-middle income settings in particular, where resources are constrained and systems at risk of fragmentation, development of the package should ensure that it works within and helps strengthen the health system, taking into account other service delivery priorities, to minimise wider unintended adverse effects.

## **Box 1**: Case study from Punjab, Pakistan

The establishment of a non-communicable disease (NCD) control Pakistan provides an example of how the ten-step process worked in a real life situation. It was based on collaboration between the Department of Health, Punjab, and an international research consortium, COMDIS-HSD, which included public health practitioner-researchers based at the University of Leeds and a non-governmental association, the Association for Social Development, based in Islamabad. The partnership has extended over 25 years, researching and developing all components of the TB programme (implemented nationally) and other chronic diseases, allowing for the development of mutually trusting relationships.

1. The scale of the problem was recognised in 2011, when the World Health Organisation (WHO) assisted the health department In Punjab to estimate the disease burden and identify wide variation in NCD care practices.
2. The Director General for Health Services Punjab formed a Technical Working Group, including COMDIS, and selected conditions for priority attention. Researchers reviewed the available WHO and other NCD literature.
3. Exploratory studies were conducted by COMDIS on these priority conditions, i.e. diabetes-hypertension and asthma-COPD. Services were found to be ad hoc clinical care only; and the focus was on how to improve access, deliver care, supervision and supplies within the district health system.
4. The package was drafted by COMDIS researchers and development team with others within the TWG. The draft products were developed according to the context, and included a case management desk guide, a counselling illustrated ‘flipchart’, record cards, training modules with case studies and communication skills role plays.
5. COMDIS pre-tested the guides and tools with a group of primary care doctors and other target users.
6. We jointly pilot-evaluated the intervention package at two health facilities, selected by the department of health NCD programme. This was a key opportunity to refine details of the desk guide, and the service delivery details outline in the package (i.e., the what, where and how of each step in the care process).
7. Trials of effectiveness were conducted; with three embedded trials i.e. diabetes-hypertension care at public rural and private urban facilities and asthma-COPD care at public facilities. All were implemented with department of health staff and resources. This was as planned and with good results, except for diabetes (insufficient time was given prior to assessing outcomes).
8. Incremental cost effectiveness analysis was also designed and conducted for diabetes-hypertension and the asthma-COPD, giving the costs of replication of the service interventions.
9. Process evaluation of the care delivery of diabetes-hypertension (at public and private facilities was) and asthma-COPD interventions were designed and conducted, assessing the loss to follow-up at each stage of the care pathway from screening onwards – and refining accordingly.
10. Scale-up was by the Department of Health, setting up and funding NCD programme with technical support by UNICEF in strategic planning and by; COMDIS in the preparation of public funding proposal, briefing documents and presentations for the programme focal person. Thereafter provincial scale-up was by the programme alone.

There were many challenges, such as ensuring district health office supply of record cards and essential drugs, as well as coping with changes in the senior department of health managers. However, the success of the ten-step process is indicated by the fact that the integrated NCD care interventions have now been scaled up during 2017–8, through public funding, to facilities within all 36 districts of Punjab, a province with a population of more than 100 million.

## Abbreviations

COMDIS-HSD: Communicable disease/Health service delivery Research Consortium
COPD: Chronic Obstructed Pulmonary Disease
NCD: Non-communicable diseases
UNICEF: UN Children’s Fund
WHO: World Health Organisation
